# Petal segmentation in CT images based on divide-and-conquer strategy

**DOI:** 10.3389/fpls.2024.1389902

**Published:** 2024-07-15

**Authors:** Yuki Naka, Yuzuko Utsumi, Masakazu Iwamura, Hirokazu Tsukaya, Koichi Kise

**Affiliations:** ^1^ Graduate School of Informatics, Osaka Metropolitan University, Sakai, Japan; ^2^ Graduate School of Science, The University of Tokyo, Tokyo, Japan

**Keywords:** CT data, petal segmentation, image segmentation, divide-conquer strategy, data augmentation

## Abstract

Manual segmentation of the petals of flower computed tomography (CT) images is time-consuming and labor-intensive because the flower has many petals. In this study, we aim to obtain a three-dimensional (3D) structure of *Camellia japonica* flowers and propose a petal segmentation method using computer vision techniques. Petal segmentation on the slice images fails by simply applying the segmentation methods because the shape of the petals in CT images differs from that of the objects targeted by the latest instance segmentation methods. To overcome these challenges, we crop two-dimensional (2D) long rectangles from each slice image and apply the segmentation method to segment the petals on the images. Thanks to cropping, it is easier to segment the shape of the petals in the cropped images using the segmentation methods. We can also use the latest segmentation method for the task because the number of images used for training is augmented by cropping. Subsequently, the results are integrated into 3D to obtain 3D segmentation volume data. The experimental results show that the proposed method can segment petals on slice images with higher accuracy than the method without cropping. The 3D segmentation results were also obtained and visualized successfully.

## 1 Introduction

Flowers have various appearances depending on their structure, size, shape, color, and number of organs that make up the flower (Shan et al., 2019; Yao et al., 2019; Bowman and Moyroud, 2024). Among the floral organs, petals are essential for understanding the flower morphology because their size, shape, and color vary widely among flower species (Huang et al., 2015) and are essential for plant reproduction (Irish, 2008) via interaction between flowers and pollinators (Whitney and Glover, 2007). Therefore, several attempts have been made to clarify the mechanism of flower morphogenesis by analyzing the shape of petals.

Most flower morphology analyses are performed by destructive examination, such as taking apart each petal one by one (Szlachetko et al., 2009; Han et al., 2019; Yao et al., 2019; Hayes et al., 2021). They have been used to clarify that petal size, shape, and genes influence flower morphogenesis. However, it is impossible to survey how petals order and develop in three-dimensional (3D) space because the flowers have been decomposed. Importantly, it is known that surface interactions between petals and other floral organs in highly packed floral bud stage can influence the final flower shape (Shimoki et al., 2021). To understand the process of petal growth and interactions among floral organs in flower bud stage, we need precise information on 3D arrangements of petals without destruction.

**Figure 1:**
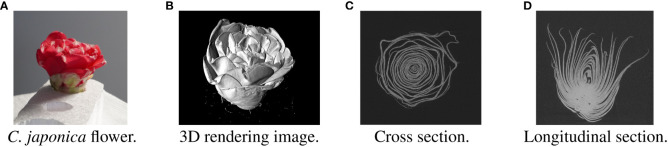
Examples of *Camellia japonica* CT volume images. **(A)** *C. japonica* flower. The image courtesy of Prof. Yutaka Ohtake, The University of Tokyo. **(B)** 3D rendering image. **(C)** Cross section. **(D)** Longitudinal section.

**Figure 2:**
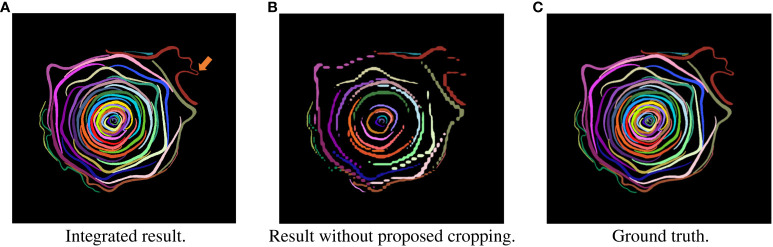
Examples of segmentation result in slice images of test data. Each petal was assigned a different color. **(A)** Integrated result. **(B)** Result without proposed cropping. **(C)** Ground truth.

**Figure 3:**
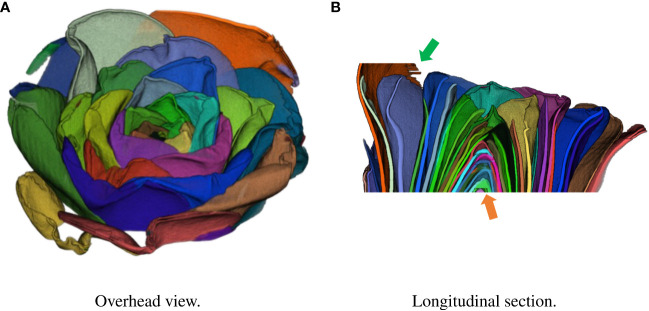
Result of integration in 3D space using test data. Each petal was assigned a different color. **(A)** Overhead view. **(B)** Longitudinal section.

In recent years, computed tomography (CT) has been used to obtain nondestructive morphological information about flowers (Hsu et al., 2017; Shimoki et al., 2021). **Figure 1** shows the *Camellia japonica* 3D volume data used in this study. **Figure 1B** rendered using the Volume Viewer, which is one of the plug-ins of Fiji is Just ImageJ (Fiji) (Schindelin et al., 2012). The data acquired by CT shows the 3D shape of the flower, but the petals are not segmented. To obtain the 3D morphological information of the petals, it is necessary to segment each petal in the CT data, as shown in **Figures 2A**, **3**. Generally, CT data segmentation is first performed on the slice images. The image segmentation results are then used to obtain the 3D segmentation results. The number of slice images is large, in the hundreds or thousands. Furthermore, segmentation is performed manually because automatic segmentation is not established. If a flower contains many petals or has a complex shape, segmenting even a single image takes a long time. Therefore, the segmentation of 3D flower data is labor-intensive, and the manual process is an obstacle to morphological analysis.

In this study, we propose an automatic segmentation method to enhance the morphological analysis of 3D flower data, focusing on *C. japonica* which is known to have great variation in petal numbers, shapes, and arrangements (Wang et al., 2021). To achieve this, how to deal with the structure of the petals, which are long and curved, is a challenge. Because the shape of the petals differs from the objects that most object detection methods deal with, they fail to detect them.

To overcome this problem, we crop rectangle regions from CT images and apply a CNN-based segmentation method to the cropped images. Cropping is performed using a long rectangle in which the center of the flower is set the center of the rectangle. We rotate the rectangle using the center of the rectangle as the rotation center and crop the images every 1° of rotation. The petals in the cropped images are less curved and appear in appearance to the object treated using the object detection methods. Therefore, conventional object detection methods can detect petals with high accuracy. After segmentation of the cropped images, the segmentation results are integrated into the 2D images, which are then integrated into the 3D data based on the intersection of union (IoU).

The experimental results show that the proposed method successfully segmented the *C. japonica* cross-sectional images and 3D data. The AP50, which are segmentation criteria, for the cropped and integrated 2D images were 0.898 and 0.900, respectively.

## 2 Related work

This section presents some examples of flower segmentation and modeling related to petal segmentation. In this study, we performed petal segmentation using CT images; therefore, we also introduce related studies on segmentation using CT images.

### 2.1 Flower segmentation

Several attempts have been made to segment flowers and petals from 2D images captured by a camera. Flower detection methods include Markov random field-based methods using graph cuts (Nilsback et al., 2010; Zagrouba et al., 2014), thresholding in the Lab color space (Najjar et al., 2012), and methods that combine HSI spatial color thresholding and local area clustering (Zeng et al., 2021). Flower segmentation methods can also be applied to agriculture, such as methods for detecting strawberry flowers (Lin et al., 2018), apple flowers (Tian et al., 2020; Sun et al., 2021; Mu et al., 2023), and tomato flowers (Afonso et al., 2019). These methods can be used to investigate special locations by detecting flowers from 2D RGB images. Therefore, these methods differ from the proposed method, which investigates the flower structure in 3D in micrometers.

### 2.2 Flower modeling

Because of the flower’s complex structure and self-occlusion, few studies have performed 3D modeling of flower shapes (Ijiri et al., 2005, 2014; Zhang et al., 2014; Hsu et al., 2017; Leménager et al., 2023). For example, Zhang et al. proposed a method (Zhang et al., 2014), in which petals are segmented from RGB images and 3D point cloud data. The segmented petals were then fitted with a pre-created morphable petal shape model to estimate the flower model. Ijiri et al. proposed a system for 3D modeling of flowers using a floral diagram, which is a schematic drawing that simply expresses the structure of the flower (Ijiri et al., 2005). Lemenager et al. used photogrammetry to obtain 3D model of flowers (Leménager et al., 2023). These methods are similar to the proposed method because they collect morphological information about flowers. However, they differ from our task because they obtain visible information that can be observed using an RGB-D camera. Our task is to obtain morphological information that cannot be observed by a camera.

Hsu et al. used micro CT data of of *Sinningia speciosa* flowers to model petals (Hsu et al., 2017). Since the flower of *S. speciosa* is a single flower, it is not necessary to apply a segmentation method does not needed to be apply before petal modeling. Ijiri et al. proposed a semiautomatic flower modeling system (Ijiri et al., 2014). To the best of our knowledge, this system is the only method for semi-automatically segmented flowers from CT images. In this system, the flowers are assumed to consist of shafts and sheets. The system defines the energy functions of the dynamic curves for the shafts and the dynamic surfaces for the sheets. Based on the energy function, the stem and petals are automatically fitted using manually specified points. This system requires considerable manual work and takes a long time to model. Therefore, it is difficult to apply this method to our task, and we should consider a fully automatic method without manual work.

### 2.3 CT image segmentation

Research on CT image segmentation has flourished in the medical field. Current studies on medical CT image segmentation primarily consist of methods that train CNNs from annotated CT image datasets. Segmentation has been used on various human organs and tissues, including the coronary arteries (Yang et al., 2020), thoracic organs (Zhou et al., 2019), ductal organs and tissues, such as blood vessels and pancreatic ducts (Wang et al., 2020), vertebrae (Masuzawa et al., 2020), and teeth (Cui et al., 2019).

Additionally, some segmentation methods (Lee et al., 2020; Yu et al., 2020; Laradji et al., 2021; Zhou et al., 2021) that focus on the affected areas. Recently, in addition to CNN, segmentation methods (Hatamizadeh et al., 2022) have been proposed that use vision transformer (ViT) (Dosovitskiy et al., 2021) for organs and brain tumors.

In computer vision research, medical image processing is recognized as one of the important research areas. Therefore, various studies have been conducted on medical CT images, as introduced above. Under such circumstances, there are many CT databases of medical images. Therefore, it is easy to apply the latest deep learning-based methods to medical CT images. However, plant image processing, especially flower image processing is not as common as medical image processing. For example, Morphosource.org^1^ contains CT images of biological specimens, including some of plants, however, their selection of flower images is limited.

## 3 Materials and methods

### 3.1 *C. japonica* flower CT data

**Figure 4:**
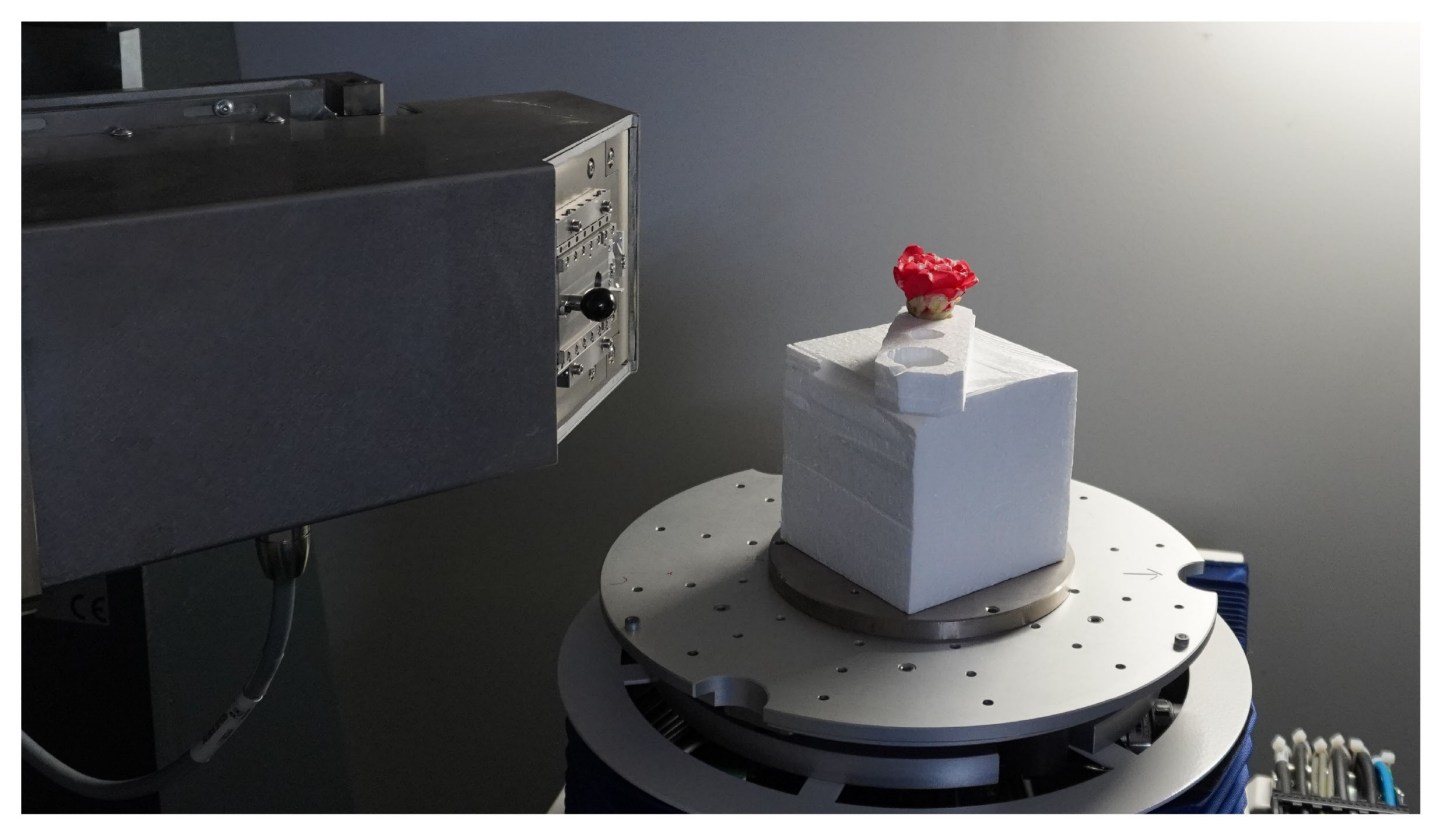
*C. japonica* CT data acquisition. Image courtesy of Prof. Yutaka Ohtake, The University of Tokyo.

We analyzed flower CT images of *C. japonica* supplied from Botanical Gardens, the University of Tokyo, Japan. ^2^ The analyzed *C. japonica* cv.”Orandako” was captured using an industrial-use dimensional X-ray CT device, METROTOM 1500 Gen.1 ^3^, made by Carl Zeiss; as shown in **Figure 4**, it was captured over 41 minutes. The X-ray tube voltage and X-ray tube current at the time of capture were 120kV and 437 mA, respectively. Other settings are shown in **Table 1**. Since the maximum time to scan the flower without changing the morphology of the flower due to drying was about 40 minutes, the settings were made so that all scans of the flower would be completed in about 40 minutes. Slice thickness is the thickness of the tomographic image in the body axis direction; in this case, this is 46.252µm.

**Table 1:** Setting values when capturing CT images.

X-ray tube voltage	120kV
X-ray tube current	437mA
Filter	None
Exposure time for each projection	1000ms
Projected image	1000 × 1000 pixels
Pixel size	0.4mm
Projections per rotation	1000
Magnification rate	8.65 times

One CT scan data was taken from one flower and used in the experiment. We created cross-sectional images shown in **Figure 1C** from the CT data and performed segmentation. The resolution of one CT image used in the analysis was 915 × 858 pixels, and there were 888 images. During the acquisition of the flower CT volume data, the flower moved slightly because it dried and changed its shape. This slight movement caused noise on the CT images. To remove the noise, we applied the nonlocal mean filter (Buades et al., 2005) with parameter *h* set to 6 before the evaluation. To train and evaluate the model, we manually assigned the ground truth for each petal at the pixel level, as shown in **Figure 2C**. We selected 39 slice images and assigned the ground truth. A total of 25 images were selected as learning data, each 10 images from the top of the flower. As test data, we selected 13 images separated by 5 slices from the training data and randomly selected one image from the slice images except for the training data.

### 3.2 Proposed segmentation method

**Figure 5:**
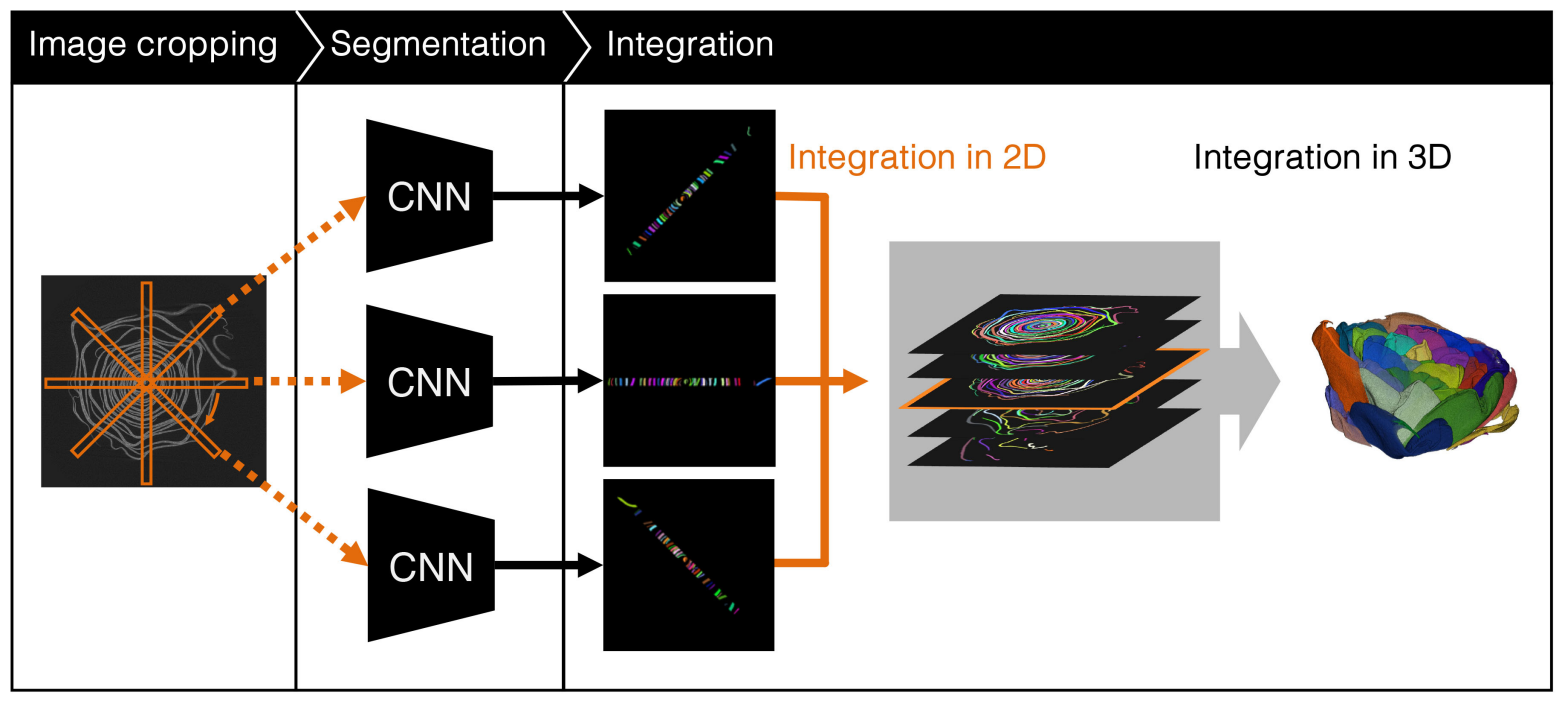
Overview of the proposed method.

An overview of the proposed method is shown in **Figure 5**. We used CT images. The proposed method crops long rectangle images from a slice image and applies a CNN-based instance segmentation method that segments each petal in a cropped image. The segmentation results in the cropped images are then integrated into the original image. Finally, the petal segmentation result is obtained by integrating the integrated segmentation images in 3D space. The following sections describe in detail the image cropping method, segmentation of the cropped images, and integration of the segmentation results.

#### 3.2.1 Image cropping

**Figure 6:**
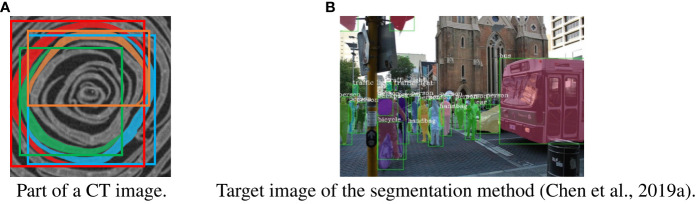
Difference between our task and general instance segmentation. **(A)** part of a slice image with petals and bounding boxes. A petal segment and its bounding box are assigned the same color. **(B)** Target image of the segmentation method. The bounding boxes are shown as green rectangles, and each instance is assigned a different color (Chen et al., 2019a).

**Figure 6** shows examples of a part of a CT image and the target image of common instance segmentation methods. As shown in **Figure 6A**, the shape of the petals is long and curved. Because of the shape, several adjacent petals appear in the bounding box of the petal. As shown in **Figure 6B**, the shape of most target objects for segmentation is approximately rectangular, and other objects do not appear in the bounding box of the object (Lin et al., 2014). Most object segmentation assumes that the shape of the target objects is similar to that in **Figure 6B**; a bounding box contains a single target object. Most object segmentation methods first detect the bounding boxes that are likely to contain the target object and then estimate the mask of the object. If there are many target objects in a bounding box, the methods will not work well because the assumption does not hold. For example, **Figure 2B** is a result when an object segmentation method is applied to our CT images, with low segmentation accuracy. Thus, object segmentation methods fail to segment petals on the CT images.

**Figure 7:**
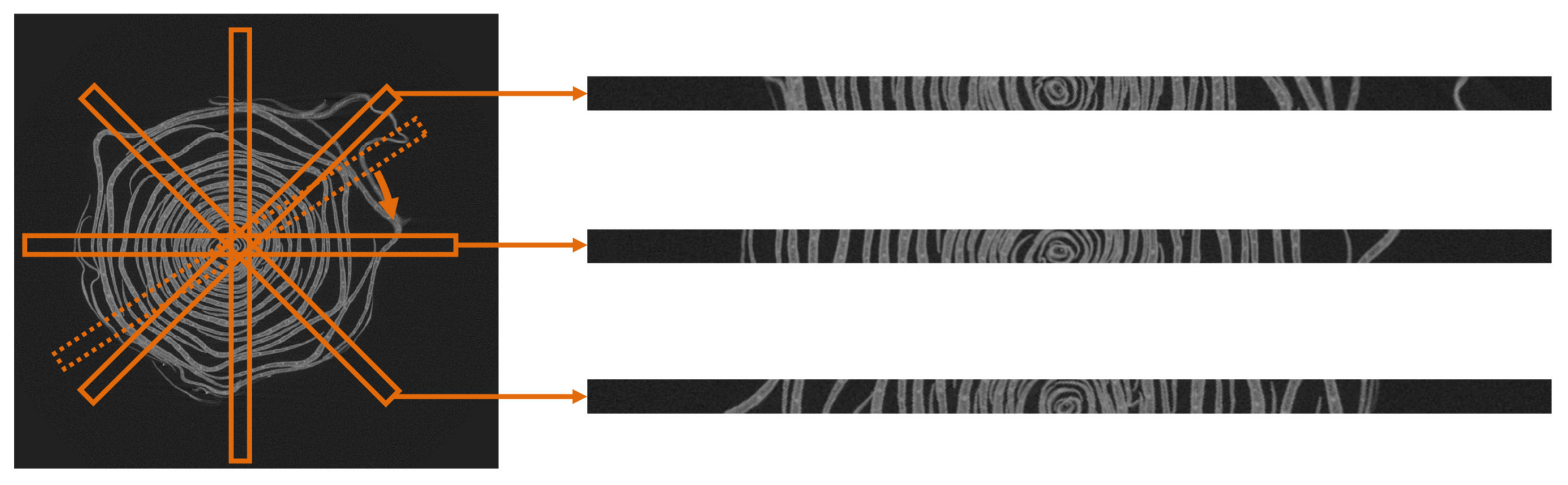
Proposed image cropping method. The orange rectangles on the left CT image show several cropped areas, and the right images show the cropped images from these areas.

To overcome the problem, we crop long rectangles from CT images and then apply the segmentation method. **Figure 7** explains how to visually crop long rectangle images. The long rectangle is set as follows: the center of the rectangle is placed in the center of the flower, and the rectangle covers both ends of the flower. We then crop long rectangles while rotating the rectangle around its center. In this study, the size of the long rectangle and the rotation interval for cropping are set to 900 × 32 pixels and 1°, respectively. As shown on the right side of the images in **Figure 7**, the petals of the cropped images are not as curved as the original slice images, and the bounding box of the petal contains only a single petal. Therefore, when the object segmentation method is applied to the cropped images, the segmentation accuracy is expected to be almost the same as that of the common target objects.

We performed the cropping using a code we wrote ourselves. The code was written in Python 3.10.8, and using OpenCV 4.9.0.80, which is an image processing library.

#### 3.2.2 Segmentation of the cropped images

After cropping the images, we apply a segmentation method to the cropped images to segment individual petals. In this study, we use the Hybrid Task Cascade (HTC) (Chen et al., 2019a) as the segmentation method. HTC is the combined model of Mask R-CNN (He et al., 2017), which is the most popular instance segmentation method in recent years and is used as the baseline for instance segmentation evaluation, and Cascade R-CNN (Cai et al., 2018), which achieves high segmentation accuracy by introducing a cascade architecture into the model. HTC won the first prize in the COCO 2018 Challenge Object Detection Task^4^. Because HTC showed high accuracy in segmentation, we decide to use it.

We used HTC implemented in MMDetection (Chen et al., 2019b), an object detection toolbox developed by OpenMMLab. MMDetection is written based on PyTorch, which is a machine learning library for Python. The version of MMDetection used in the experiment is v2.28.2^5^.

#### 3.2.3 Integration of the segmentation results

After obtaining the segmentation results in the cropped images, we perform integration to obtain the 3D segmented volume data. We first integrate the results into the slice images and then integrate the slice images into the 3D volume.

**Figure 8:**
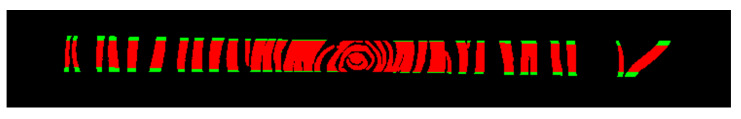
Difference between the two cropped images with a rotation interval of 1°. The red and green areas show the overlapping and nonoverlapping areas of the petals of the two cropped images, respectively.

We integrate the segmentation results using the overlapping regions in the cropped images. When the cropped images are set to the cropped locations, there is an overlap between the adjacent cropped regions. **Figure 8** shows the overlapping petal regions between the cropped images with a rotation interval of 1°. We consider the segmentation results in the overlapping regions to be the same petals, and then integrate the segmentation results.

**Figure 9:**
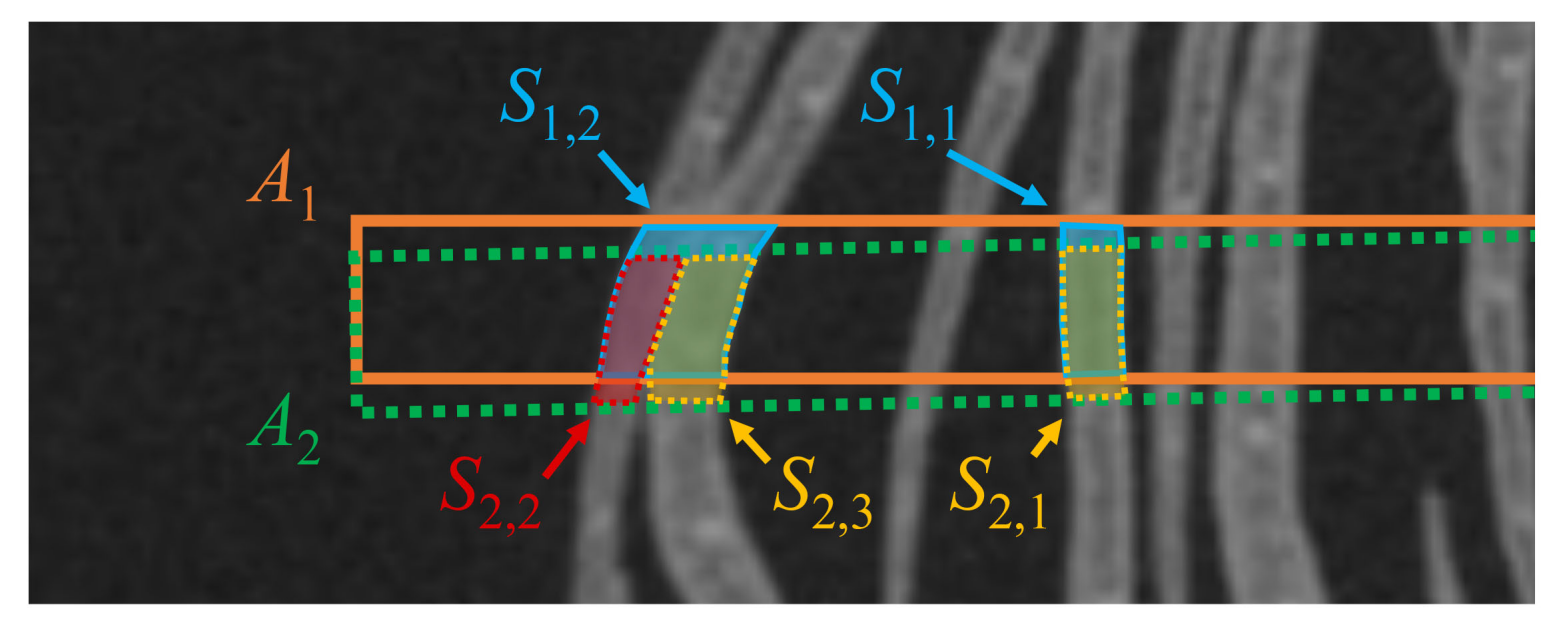
Example of two petal segmentation results in two adjacent cropped images. The orange and green dotted rectangles show the cropped regions *A*_1_ and *A*_2_. *S*_1_,_1_, *S*_1_,_2_ and *S*_2_,_1_, *S*_2_,_2_, *S*_2_,_3_ are the segmentation results for *A*_1_ and *A*_2_, respectively.

The segmentation results contain errors because they are not always accurate. Errors cause the integration to fail. To avoid integration failure, we remove errors before integration. Suppose that the segmentation results shown in **Figure 9** are given. *A*_1_ and *A*_2_ are adjacent cropped images, and *S*_1_,_1_, *S*_1_,_2_ and *S*_2_,_1_, *S*_2_,_2_, *S*_2_,_3_ are the segmentation results on *A*_1_ and *A*_2_, respectively. If there is a disagreement between the segmentation results in the overlapping regions, we consider the segmentation result that is divided into more regions as correct. Then, another segmentation result is considered incorrect, and is removed from the segmentation results. As shown in **Figure 9**, because the overlapping area of *S*_1_,_2_ is segmented into *S*_2_,_2_ and *S*_2_,_3_ on *A*_2_, *S*_1_,_2_ is considered to be an incorrect segmentation result, and *S*_1_,_2_ is removed from the segmentation results.

After removing the errors, we integrated the segmentation results. We use IoU as the integration criterion. IoU indicates the degree of overlap between the two regions. Let *A* and *B* be the two regions on an image, and | · | be the number of pixels in the region. The IoU between *A* and *B* is obtained by the Equation 1, as follows:


(1) $$I\text{oU}=\frac{\left|A\cap B\right|}{\left|A\cup B\right|}$$

We calculate the IoU of overlapping segmentation results and integrate the pair of segmentation results that yield an IoU of 0.8 or more.

To obtain 3D segmentation results, the 2D integrated images are integrated into 3D. The integration in 3D is to stack the 2D integrated images. However, since the segmentation in each 2D image is performed independently, 3D segmented data is not obtained by simply stacking the 2D images. Therefore, we determine which regions are from the same petal based on the IoU for the adjacent frames as the 2D image integration. We calculate the IoUs between a segment of a 2D integrated image and all segments of the adjacent image. The pair that gives the highest IoU and whose value is equal to or greater than 0.8 is considered the same petal and is integrated. This process is applied sequentially starting from the top frame to obtain the 3D integration of the segmentation result.

For 2D and 3D integration, we used code we developed ourselves. The code uses Python 3.10.8 and OpenCV 4.9.0.80. After performing the integration, regions identified as the same petals were colored consistently in the final result. We visualized the segmentation results of the 3D volume by displaying the final result images using Fiji’s Volume Viewer.

## 4 Experiment

To evaluate the proposed method, we applied it to the CT data introduced in Subsection 3.1.

### 4.1 Experimental setting

The data used to train the model were 25 CT images with manually annotated petal regions. The 900×32 pixel images were cropped using the proposed method described in Subsection 3.2.1. Since the images were cropped every rotation of 1°, the number of cropped images from a training image was 360, and the total number of cropped images was 9000. The evaluation data consist of 14 manually annotated images which are from the same CT data but different from the training data. We also cropped images from the evaluation data using the proposed method and prepared a total of 5040 images. We used nearest-neighbor interpolation when cropping the images. We filled the empty pixels with random values with a normal distribution. The mean was the average of the background pixel values, and the variance was 5. We also applied the proposed method to the CT images, except for the evaluation data, and integrated the segmentation results in 3D.

**Figure 10:**
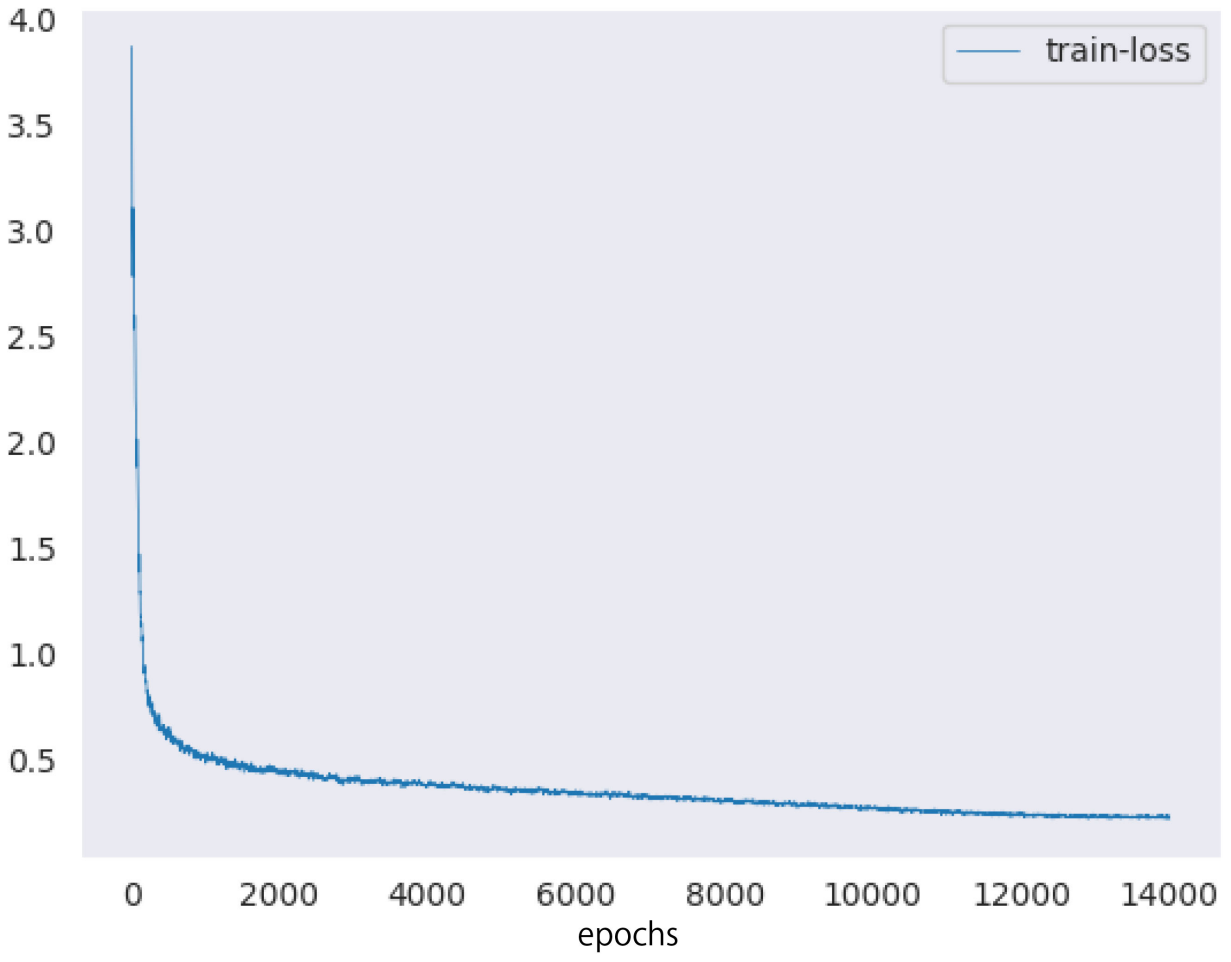
The training loss of the proposed method.

Next, we describe the setup of the HTC model. We used ImageNet (Deng et al., 2009) pretrained ResNeXt-101 (Xie et al., 2017) 64×4*d* and the feature pyramid network (Lin et al., 2017) as the backbones of the HTC model. The number of epochs and batch size were set to 20 and 32, respectively. We used the AdamW optimizer (Loshchilov et al., 2019) as the optimization algorithm to train the model. The training rate was reduced from 10^−4^ to 10^−7^ using the cosine annealing scheduler (Loshchilov et al., 2017). **Figure 10** showed the training loss of the proposed method.

We used COCO API^6^ to calculate the accuracy of the results. We used precision and recall to evaluate the petal segmentation results. We calculated the IoUs between each segmentation result and its ground truth and considered the segmentation results whose IoU was greater than a threshold as correct. The average precision (AP) and average recall (AR) were calculated when the IoU threshold was 0.5 and 0.75, denoted AP_50_, AR_50_, AP_75_, and AR_75_, respectively. We also calculated the mean average precision and recall (mAP and mAR), which are the mean AP and AR when the IoU threshold was changed from 0.5 to 0.95 with a 0.05 interval.

In addition to evaluating the proposed method, we evaluated the segmentation accuracy using RandAugment, which is one of the popular augmentation methods, instead of the proposed cropping method. We trained the model using 25 training images, which are the same as the training data for the proposed method, without cropping. The training data was augmented by RandAugment (Cubuk et al., 2020). The number of augmentation transformations and magnitude for all the transformations are set to 4 and 10, respectively. The data augmentation we used were rotation, horizontal and vertical translation, and flip. The number of epochs and batch size were set to 50 and 16, respectively, and the other parameters were the same as that used in the experiment of the proposed method. The model was also evaluated using 14 images, which are the same evaluation data as the proposed method. To compare the accuracy with the proposed method, we calculated AP_50_, AR_50_, AP_75_, AR_75_, mAP, and mAR of the segmentation results.

We used a GPU server for model training and a CPU server for segmentation and integration. The GPU server was equipped with an NVIDIA TITAN RTX and 24 GB of memory. The CPU server was equipped with an Intel Xeon Gold 5118 processor and 128 GB of RAM.

To evaluate the proposed method using morphological properties, we calculated the estimation error of the petal area on a CT image using three test images. We manually matched the segmented regions with the ground truth, then calculated the area by counting the pixels in each region. The error between the ground truth and the detected regions was calculated for each petal, and the pixel count was converted into area. Given that each pixel side is 46.252µm, the area of each pixel is 46.252×46.252 = 2.1392×10^3^ µm^2^. The mean and variance of the petal areas obtained from the images were 5.58mm^2^ and 3.52mm^2^, respectively. Due to the large variation in petal areas indicated by the mean and variance, we used the mean absolute percentage error (MAPE) to evaluate the area estimation error. Assuming *n* is the total number of petals, *E_i_* is the area of the *i*th estimated petal, and *G_i_* is the area of the ground truth for the *i*th petal, the MAPE is calculated by Equation 2, as follows:


(2) $$\text{MAPE}=\frac{100}{n}\sum_{i=1}^{n}\left|\frac{E_{i}-G_{i}}{G_{i}}\right|.$$

### 4.2 Results

**Figure 11:**
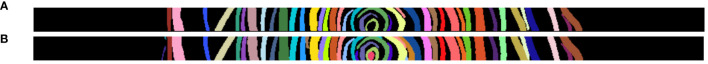
Examples of segmentation results in cropped images of test data: **(A)** segmentation results and **(B)** ground truth. Each petal was assigned a different color.

First, we evaluated the segmentation results of the cropped images. **Table 2** shows the scores of the accuracy evaluation metrics for the cropped images. AP_50_ and AR_50_ were 0.898 and 0.906, respectively. **Figure 11** shows examples of the segmentation results for the cropped images.

**Table 2:** Petal segmentation results on the cropped images.

Metric	mAP	AP_50_	AP_75_	mAR	AR_50_	AR_75_
Score	0.585	0.898	0.683	0.624	0.906	0.727

Next, we evaluated the integrated images and compared the results with the segmentation results without cropping. **Table 3** shows the scores of the accuracy evaluation metrics for the integrated images and the segmentation results without cropping. AP_50_ and AR_50_ of the proposed method were 0.900 and 0.928, respectively. **Figure 2** shows examples of the integrated and segmentation results without cropping. In the evaluation of morphological properties, the MAPE was 7.25%.

**Table 3:** Petal segmentation results after integration and without cropping.

	mAP	AP_50_	AP_75_	mAR	AR_50_	AR_75_
Integration	0.714	0.900	0.804	0.781	0.928	0.871
w/o cropping	0.214	0.606	0.073	0.277	0.635	0.199

Finally, we show the integration result in 3D space. **Figure 3** shows the segmentation volume data and its longitudinal section after 3D integration. Volume data were rendered using the Volume Viewer of Fiji. We also show a video of the segmented 3D volume data in the **Supplementary material**.

## 5 Discussion

In the segmentation of the cropped images, the results of AP_50_ and AR_50_ show a good level of accuracy. By performing the cropping, the bounding box of a petal contains the petal only, as in general segmentation data such as MS COCO (Lin et al., 2014). This would have improved the estimation accuracy of the petal region. **Figure 11** shows that segmentation is generally successful, although some petals are missing or inaccurately segmented in complex areas such as the center.

As shown in **Table 2**, the values of AP_75_, AR_75_, mAP, and mAR are much lower than those of AP_50_ and AR_50_. This is because of the size of the petal regions: the median petal area in the cropped image for evaluation was 223 pixels, and most of the petal area was less than 400 pixels. Generally, the IoU is sensitive to misalignment. In particular, when the size of the area to be calculated for the IoU is small, the IoU drops drastically even if a 1-pixel misalignment occurs. Therefore, the IoU threshold of 0.5 for determining the segmentation success is considered sufficient to evaluate the accuracy, and AP_50_ and AR_50_ are the appropriate criteria for segmentation in this task.

As shown in **Table 3**, the integrated segmentation results show better accuracy for all metrics. This is because of the removal of incorrect segmentation results before integration. Thanks to the removal, the integration was successful and showed better accuracy. The IoU property is also responsible for accuracy. Additionally, the IoU was less sensitive than that in the cropped images because the area of the petals was larger than that in the cropped images (Zheng et al., 2020). As in the case of cropped images, segmentation is generally successful, although there are some errors in the central position in **Figure 2**. The orange arrow in **Figure 2A** indicates the area where segmentation failed. This is because the boundary between the two petals was ambiguous and could not be divided into petals.

Compared with the integrated segmentation results and the segmentation results without the proposed cropping method (Chen et al., 2019a), the integrated segmentation results quantitatively and qualitatively exceed the results without the proposed cropping method, as shown in **Table 3** and **Figures 2A, B**. This shows that the proposed cropping method is effective for petal segmentation.

The segmentation of 3D volume data by 3D integration was generally successful as shown in **Figure 3A**. As shown in **Figure 3B**, the center of the flower indicated by the orange arrow was incorrectly segmented. This is a limitation of the segmentation model because the segmentation of the center failed in the cropped images. The edge of a petal indicated by the green arrow was also incorrectly segmented because of noise in the data. A different denoising method such as CNN-based method (Zhang et al., 2021) can lead to successful segmentation. In addition, the limited training data may have decreased the accuracy. Increasing the training data would improve the segmentation accuracy in 2D slice images and thus improve the integrated 3D segmentation result.

A limitations of this research is that the training and test data were derived from the same CT data. If the training and test images had come from different CT data, the segmentation accuracy might decreased. The algorithm for eliminating detection errors before integrating the segmentation results of cropped images performed well in this experiment. However, if the segmentation accuracy declines, the algorithm’s performance would suffer, leading to a decrease in the accuracy of the integrated results. In such cases, increasing the amount of training data or revising the integration algorithm might be necessary to enhance the accuracy of the segmentation results.

The other limitation is the CT scanning setting. Usually, CT scans of flowers are conducted with water and in a controlled humidity environment. Our scanning setting is unconventional, making it uncertain the flower shape is captured as accurately as with the usual method. However, our proposed segmentation method has shown sufficient accuracy with our data. Therefore, if the CT scans are performed with water and controlled humidity, and our proposed method is applied, we expect to obtain highly accurate 3D shapes with segmented petals.

## 6 Conclusion

This paper proposed a petal segmentation method for *C. japonica* flower CT images. It is difficult to manually segment each petal on the CT images because the number of CT images from the volume data and the number of petals to be segmented on the slice images are large. Therefore, we automatically segmented the petals on the slice images using machine learning based image recognition techniques. To overcome the decrease in segmentation accuracy due to the shape of the petals, we crop the long rectangle images from the slice images and apply the latest segmentation method. Consequently, 3D segmentation results were obtained by integrating the segmentation on the cropped images. The experimental results showed that the proposed method outperformed the method without cropping images in terms of segmentation accuracy. Moreover, we successfully segmented 3D flower volume data by integrating the segmentation results.

## Data availability statement

The datasets presented in this study can be found in online repositories. The names of the repository/repositories and accession number(s) can be found below: https://doi.org/10.6084/m9.figshare.25264774.v1. The code implementing the proposed method is available at https://github.com/yu-NK/petal_ct_crop_seg.git.

## Author contributions

YN: Data curation, Formal analysis, Investigation, Methodology, Software, Validation, Visualization, Writing – original draft. YU: Conceptualization, Funding acquisition, Methodology, Project administration, Supervision, Writing – original draft, Writing – review & editing. MI: Methodology, Supervision, Writing – review & editing. HT: Conceptualization, Data curation, Supervision, Writing – review & editing. KK: Supervision, Writing – review & editing.

## Footnotes

1
https://www.morphosource.org.

2 The dataset is available from https://doi.org/10.6084/m9.figshare.25264774.

3
https://www.zeiss.co.jp/metrology/seihin/shisutemu/ct/metrotom.html.

4
https://cocodataset.org/workshop/coco-mapillary-eccv-2018.html.

5 HTC model implemented in MMDetection is available from https://github.com/open-mmlab/mmdetection.git .

6
https://github.com/cocodataset/cocoapi.
